# Biomonitoring: a demonstration of the importance of direct analytical methods for the assessment of chemical exposures

**DOI:** 10.3389/fchem.2026.1894102

**Published:** 2026-09-03

**Authors:** Roy Gerona, Alia Sovereign, Patricia Hunt

**Affiliations:** 1 Department of Obstetrics, Gynecology and Reproductive Sciences, Center for Reproductive Sciences, University of California, San Francisco, San Francisco, CA, United States; 2 School of Molecular Biosciences and Center for Reproductive Biology, Washington State University, Pullman, WA, United States

**Keywords:** bisphenol S (BPS), direct method, endocrine-disrupting chemicals, LC-MS/MS, monobutyl phthalate, propylparaben

## Abstract

**Introduction:**

Biomonitoring of commercial endocrine-disrupting chemicals (EDCs) increasingly underpins exposure assessment and risk evaluation, yet most large-scale studies still rely on indirect, enzymatic hydrolysis–based methods whose quantitative performance has not been systematically verified. Here, we describe the development and rigorous validation of a direct liquid chromatography-tandem mass spectrometry (LC-MS/MS) assay using validated standards to simultaneously quantify bisphenol S (BPS), propylparaben (PrP), monobutyl phthalate (MBP), and their major urinary glucuronide and sulfate conjugates in human urine.

**Methods:**

A direct LC–MS/MS assay was developed for the simultaneous quantification of BPS, PrP, MBP and their major urinary metabolites. The method was validated in accordance with U.S. Food and Drug Administration bioanalytical guidelines, including assessments of linearity, accuracy, precision, selectivity, specificity, matrix effects, recovery, and carryover. The validated assay was then used to evaluate the accuracy of a conventional β‐glucuronidase–based hydrolysis workflow across a wide concentration range in spiked synthetic urine by comparing indirect measurements with direct totals for each analyte. Method utility in human biomonitoring was demonstrated by analysis of urine samples from 30 pregnant women in their second trimester.

**Results:**

Using isotope-dilution calibration, solid-phase extraction, and negative-ion electrospray multiple reaction monitoring, the method achieved sub‐ng/mL limits of detection for all analytes, linear response over 3-4 orders of magnitude, and intra‐ and inter‐day precision and accuracy within contemporary bioanalytical criteria, confirming fitness for trace-level biomonitoring. Indirect, hydrolysis-based measurements closely tracked direct totals for BPS and PrP, but systematically underestimated MBP, with a concentration-dependent negative bias that increased at higher levels, demonstrating that hydrolysis efficiency is analyte-specific and cannot be inferred from surrogate substrates alone. Application of the direct method to archived urine samples from 30 pregnant individuals enabled the first simultaneous resolution of the free and conjugated forms of these three EDCs in a maternal cohort and revealed that glucuronides accounted for the majority of the total urinary burden, with total concentrations exceeding contemporary NHANES estimates.

**Discussion:**

Collectively, these findings show that indirect methods can introduce substantial, analyte-dependent underestimation of internal dose, with implications for exposure misclassification, attenuation of epidemiologic effect estimates, and underestimation of population risk. The data support the position that direct LC–MS/MS quantification of parent and conjugated species, coupled with analyte-resolved assessment of hydrolysis efficiency, should become standard practice in method validation and national biomonitoring programs to ensure accurate exposure assessment for non‐persistent EDCs.

## Introduction

Analytical precision and specificity are central to environmental health risk assessment, informing regulatory toxicology, public health policy, and risk communication. The traditional risk framework links hazard identification with dose–response characterization and estimates of human exposure levels. Because exposure estimates based on biomonitoring data serve as the primary readout of contaminant burden in human populations (Environmental Protection Agency, 2025), the performance of biomonitoring methods directly affects the reliability of risk evaluations.

As an academic analytical laboratory, we specialize in two types of quantitative measurements in human biospecimens: street drugs and environmental chemical contaminants used in products. Although these two forms of biomonitoring appear superficially similar, in practice, they differ substantially in regulatory context, availability of reference materials, and complexity of exposure pathways. Drug biomonitoring typically benefits from extensive federal support, well-characterized metabolism, and rapid development of reference metabolite standards, whereas analogous information for manufactured chemical contaminants is often incomplete and reference standards emerge more slowly, typically after evidence of hazard is published. In addition, pervasive background contamination poses persistent challenges for trace-level quantification of commercial chemicals used in products without prior hazard testing (Matouskova et al., 2023). Given these differences, the development, tailored implementation, and rigorous evaluation of analytical strategies to ensure accurate measurement of manufactured chemical contaminants are essential.

Biomonitoring is a relatively young component of analytical chemistry, and many needs and constraints specific to commercial chemical contaminants remain underappreciated. Here, we focus on biomonitoring for endocrine-disrupting chemicals (EDCs), which mimic or interfere with endogenous hormonal signaling and are present in a wide range of products and environments (soil, water, air). Our data highlight the limitations of commonly used indirect analytical methods and introduce a direct liquid chromatography–tandem mass spectrometry (LC–MS/MS) method. This direct approach provides a means of assessing the accuracy of currently indirect hydrolysis-based methods (Blount et al., 2000; Ye et al., 2008; Zhou et al., 2014).

### Ubiquitous chemical contaminants in consumer products: a new frontier in analytical chemistry

Since the 1990s, advances in analytical chemistry, particularly mass spectrometry-based techniques, have substantially improved the ability to quantify trace manufactured chemical contaminants in complex biological matrices. LC–MS/MS has become the workhorse platform because it provides high sensitivity, analyte selectivity, and the capacity to measure multiple compounds and metabolites in a single run. The adaptation of LC-MS/MS methods coincided with growing concern about the biological effects of EDCs (Colborn et al., 1992; Kavlock et al., 1996). Data from experimental studies suggest a variety of adverse exposure effects, including metabolic, neurological, cardiovascular, reproductive, and developmental effects (Colborn et al., 1993; Gore et al., 2015; Kumar et al., 2020). The widespread use of EDCs in manufacturing and consumer products has led to ubiquitous low-level exposure in the general population, and large-scale programs, e.g., the U.S. National Health and Nutrition Examination Survey (NHANES), have incorporated EDC biomonitoring to determine exposure levels in human populations.

Regulatory decisions regarding EDCs have proved problematic because these chemicals challenge an assumption inherent in risk assessment: that the association between exposure level and elicited adverse effects will be linear. EDCs, like hormones, can exhibit nonmonotonic dose–response relationships (Vandenberg et al., 2012), making it difficult to define thresholds for safe exposure. Indeed, given their hormone-like behavior, it has been argued that there is no safe level of exposure for EDCs (Vom Saal and Welshons, 2026).

EDCs are diverse, with wide-ranging differences in physicochemical properties and environmental persistence. Hydrophobic EDCs, like polychlorinated biphenyls (PCBs), per- and polyfluoroalkyl substances (PFAS), and polycyclic aromatic hydrocarbons (PAHs), tend to accumulate in adipose tissue and display long biological half-lives. In contrast, moderately hydrophilic, non-persistent EDCs, including bisphenols, parabens, and phthalates, undergo rapid phase II metabolism (primarily glucuronidation and sulfation) and are excreted predominantly as conjugated metabolites in urine (Abbas et al., 2010; Calafat et al., 2007; Frederiksen et al., 2007; Vom Saal et al., 2014).

Human urine is the most commonly used matrix for biomonitoring of non-persistent EDCs because it is non-invasive to collect and reflects recent exposure. However, many target analytes in urine are present almost exclusively as phase II conjugated metabolites, and until recently, reference standards for these conjugates were limited. In the absence of such standards, biomonitoring has typically relied on “indirect”, LC-MS/MS workflows in which enzymatic hydrolysis (e.g., with β-glucuronidase) is used to deconjugate metabolites and “total” analyte levels are inferred by quantifying the liberated parent unconjugated compound. This approach assumes complete and reproducible deconjugation and equivalence between the released aglycones and the sum of its conjugate forms (Caballero-Casero et al., 2016; Vandenberg et al., 2010). These assumptions have not been tested directly for many analytes, and may not hold across laboratories, matrices, or enzyme conditions.

Recent increases in the commercial availability of conjugated metabolite standards have enabled the development of “direct” analytical methods that chromatographically resolve and simultaneously measure parent compounds and their glucuronide and sulfate conjugates without prior hydrolysis. Early work for bisphenol A (BPA) has shown that such methods can achieve good inter-laboratory reproducibility and have been applied in biomonitoring studies (Battal et al., 2014; Gerona et al., 2013; Gerona et al., 2016; Hauck et al., 2016; Li et al., 2022; Liao and Kannan, 2012; Nachman et al., 2015; Provencher et al., 2014; Vandenberg et al., 2014). Emerging data from our laboratory suggest that indirect hydrolysis-based approaches underestimate urinary BPA concentrations (Gerona et al., 2020), raising concerns that similar biases may also affect other phenols and phthalates, which are still quantified almost exclusively by indirect methods. However, systematic, analyte-specific comparisons between indirect and direct approaches across multiple non-persistent EDCs remain limited. Our data presented here and elsewhere show that while the use of a surrogate standard and indirect enzymatic hydrolysis provides repeatable results (they are reproducible), the results are not accurate (they are not valid).

To analyze representative chemicals from three different classes of non-persistent EDCs, we focused on bisphenol S (BPS), propylparaben (PrP), and monobutyl phthalate (MBP) and their major urinary conjugates. These three analytes are widely used in consumer products, routinely detected in human urine, and included in large biomonitoring efforts, including NHANES and several pregnancy cohorts, where they show high detection frequencies (typically >80–90% of participants for BPS, PrP, and MBP in survey cycles). At the same time, they span structurally and physicochemically distinct subclasses (bisphenol analogues, cosmetic preservatives, and phthalate metabolites) that all undergo extensive glucuronidation and/or sulfation, but with differing conjugate profiles and enzyme–substrate characteristics. This combination of broad biomonitoring relevance, high detection frequency in national surveys, and mechanistic diversity renders BPS, PrP, and MBP a stringent and informative test panel for evaluating the performance and analyte-specific bias of direct versus indirect hydrolysis-based urinary EDC methods.

We report here the development and validation of a direct LC–MS/MS method for the simultaneous quantification of BPS, PrP, MBP, and their principal conjugated metabolites in human urine. Following evaluation of key performance characteristics, including selectivity, specificity, sensitivity, linearity, accuracy, precision, matrix effects, and recovery, we used the method to evaluate the accuracy of conventional indirect enzymatic hydrolysis-based methods. A comparison of results obtained when both direct and indirect methods were applied to the same spiked synthetic human urine samples allowed us to quantify the extent and direction of discrepancies between methods for these non-persistent EDCs. Our aim was to characterize the analytical bias associated with hydrolysis-based approaches and assess how such bias may influence estimates of population exposure and associated risk metrics.

## Materials and methods

### Chemicals and reagents

Authentic standards of BPS, BPS sulfate, BPS glucuronide, MBP, MBP glucuronide, PrP, and PrP glucuronide were obtained from commercial suppliers, including Toronto Research Chemicals, Santa Cruz Biotechnology, and Sigma Aldrich. Isotopically labeled internal standards (BPS-^13^C_12_, BPS glucuronide-^13^C_12_ sodium salt, MBP-d4, PrP-^13^C_6_) were sourced from the same vendors. HPLC-grade water and ammonium acetate were purchased from Fisher Scientific, LC–MS grade methanol from VWR International, and synthetic human urine (SHU) from UTAK Laboratories. β-Glucuronidase (Type H-3, Helix pomatia, 90,000 units/mL) was obtained from Sigma Aldrich.

### Sample preparation

Calibration standards (0.02–250 ng/mL) and quality control (QC) samples were prepared in SHU by serial dilution of mixed working solutions, with internal standards added to each level. Low- and high-QC samples were made from independent stock solutions. Urine specimens from the second trimester of pregnancy were analyzed by both direct and indirect methods. For indirect analysis, 250 µL urine was acidified (pH 4.5), incubated with β-glucuronidase (450 U/reaction mixture) at 37 °C for 2 h, then treated with methanol to deactivate the enzyme before internal standard addition. For direct analysis, 250 µL urine was spiked only with the internal standard mixture. Commercially available isotopologues of the target analytes were used as internal standards. All calibration, QC, and sample extracts underwent solid-phase extraction (SPE) using Bond Elut Plexa (30 mg, 1 cc, Agilent Technologies), followed by evaporation at 32 °C, reconstitution in 250 µL 90:10 water: methanol, and centrifugation at 3,000 rpm for 10 min prior to LC–MS/MS analysis. When urine samples require dilution for quantification, they are diluted with synthetic human urine to maintain matrix matching between samples, calibration standards, and QC materials.

### Instrumentation and LC–MS/MS analysis

Chromatographic separation was performed on an Agilent 1260 HPLC coupled to an AB Sciex 5500 triple quadrupole mass spectrometer, using electrospray ionization in negative mode. The Zorbax Extend-C18 column (4.6 × 100 mm, 1.8 µm, with guard column) provided separation of analytes. Mobile phase A was water with 0.05% ammonium acetate (pH ∼7.8), and mobile phase B was methanol with 0.05% ammonium acetate. The gradient profile progressed from 30% B (0–0.5 min) to 65% B (0.5–4 min), held until 5 min, ramping to 70% B (5–7 min), 100% B (7–12 min), then returning to 30% B (12–15 min), at a flow rate of 0.70 mL/min. Injection volume was 25 μL; column oven set at 40 °C; autosampler at 4 °C. Mass spectrometric acquisition was performed in negative electrospray ionization (ESI) mode, using multiple reaction monitoring (MRM). Quantifier and qualifier transitions, including compound-dependent parameters used to generate them, are summarized in Table 1.

**TABLE 1:** MRM transitions, instrument settings (compound-dependent parameters) and internal standard used for each target analyte in the panel.

Analyte	Q1*	Q3*	Dwell time (ms)	DP* (V)	EP* (V)	CE* (V)	CXP* (V)	Internal standard used
BPS^13^C_12_	261.0	114.0	21	−100	−10	−36	−16	​
BPS-G^13^C_12_	437.1	261.0	21	−45	−10	−38	−21	​
PrP^13^C_6_	185.0	98.0	21	−5	−10	−30	−9	​
MBP d4	225.0	81.0	21	−45	−10	−24	−7	​
BPS (Quant)	249.1	108.0	21	−75	−10	−34	−9	BPS^13^C_12_
BPS (Qual)	249.1	91.9	21	−75	−10	−40	−11	​
BPS-G (Quant)	425.0	248.9	21	−80	−10	−38	−19	BPS-G^13^C_12_
BPS-G (Qual)	425.0	107.9	21	−80	−10	−64	−9	​
BPS-S (Quant)	328.9	248.9	21	−5	−10	−30	−19	BPS-G^13^C_12_
BPS-S (Qual)	328.9	107.9	21	−5	−10	−52	−11	​
PrP (Quant)	179.1	92.0	21	−50	−10	−28	−7	PrP^13^C_6_
PrP (Qual)	179.1	136.0	21	−50	−10	−20	−11	​
PrP-G (Quant)	355.0	112.9	21	−135	−10	−18	−11	PrP^13^C_6_
PrP-G (Qual)	355.0	179.0	21	−135	−10	−30	−15	​
MBP (Quant)	221.2	71.0	21	−30	−10	−16	−11	MBP d4
MBP (Qual)	221.2	68.9	21	−30	−10	−16	−33	​
MBP-G (Quant)	397.1	221.0	21	−50	−10	−16	−19	MBP d4
MBP-G (Qual)	397.1	175.0	21	−50	−10	−14	−15	​

* Q1, parent ion; Q3, fragment ion; DP , declustering potential; EP , exit potential; CE , collison energy; CXP , collision exit potential; Qual and Quant in the analyte designations mean Qualitative transition and Quantitative transition, respectively.

### Data analysis and quantification

Peak detection and integration were performed using Analyst 1.7.2 and MultiQuant 2.1 software (AB Sciex), respectively. Quantification relied upon isotope dilution with corresponding isotopically labeled internal standards. When an isotopologue of the analyte was unavailable, the internal standard with the closest retention time or chromatographic behavior to the analyte was used. Signal-to-noise (S/N) ratios, retention times, and transition ion ratios were used for confirmation.

### Method validation

Validation included selectivity, specificity, linearity, sensitivity, precision, accuracy, recovery, matrix effect, and carryover, in accordance with US Food and Drug Administration bioanalytical guidance (Food and Drug Administration, 2018).

#### Selectivity and specificity

Selectivity was evaluated by comparing chromatograms and mass spectra from blank SHU extracts with SHU samples spiked at the LLOQ and higher QC levels to confirm the absence of interfering peaks at analyte retention times and transitions. Specificity was assessed by adding structurally related compounds (bisphenol A, bisphenol F, bisphenol AF, selected phthalate metabolites, and other parabens) and verifying that they did not generate a significant signal in the analyte transitions or distort the analyte peak area or retention time.

#### Linearity and sensitivity

Linearity was established using five full calibration curves on three independent days, applying linear regression with back calculated standards within ±15% of spiked value. Sensitivity was characterized by determining LOD as the lowest standard with S/N > 3 and LLOQ as the lowest standard with S/N > 10, r^2^ > 0.95, and acceptable precision and accuracy. These criteria ensured reliable quantification across the intended concentration range, particularly at low exposure levels.

#### Precision, accuracy, and injection reproducibility

Precision and accuracy were evaluated using five replicate low and high QC samples in both intra day and inter day runs. Precision was expressed as coefficient of variance, “CV” (%) = “SD”/“mean” ×100, and accuracy as relative error, “RE” (%)=(“mean” -“nominal”)/“nominal” ×100. Acceptance required CV and RE within ±15% at QC levels. Injection reproducibility was assessed by five consecutive reinjections of the same low and high QC samples, confirming stable peak area and retention time within the same CV limits.

#### Recovery and matrix effects

Recovery was determined by comparing analyte responses in pre extraction spiked SHU with post extraction spiked SHU at matched concentrations, and expressing percent recovery as the ratio of these responses × 100. Consistent recovery with low CV across levels indicated efficient and reproducible extraction. Matrix effects were assessed by comparing post extraction spiked SHU with neat solvent spiking; percent matrix effect was calculated as the ratio of post extraction to neat responses × 100. The acceptance criteria set for both matrix effect and recovery was based on their precision (CV within 15%).

#### Carryover

Carryover was evaluated by injecting SHU blanks immediately after high QC samples at the upper calibration range. Residual analyte signal in blanks was required to be ≤ 20% of the LLOQ response. The absence of significant residual peaks confirmed that the injection and washing protocol effectively minimized carryover and that high concentration samples did not materially affect subsequent measurements.

### Comparison of direct and indirect methods

To evaluate method performance, six sets of spiked SHU samples (0.5–500 ng/mL) were analyzed via both indirect (enzymatic hydrolysis) and direct methods, in triplicate across three separate days. Direct and indirect quantification results were statistically compared and assessed for recovery bias.

### Human urine sample analysis

Thirty urine samples from second-trimester pregnant women (collected in Northern and Central California between 2011 and 2014) were analyzed using the validated direct method for all target analytes and major conjugates. Each sample was injected in duplicate, and the mean of the two replicate measurements was used as the reported concentration. To ensure the validity of data from each analytical run, two procedural quality control materials (low and high) were analyzed in each batch, along with procedural blanks and calibration curves placed at the beginning, middle, and end of each batch. Batch results were accepted only if QC measurements were within 15% of their target values, as well as within-run precision within 15% coefficient of variation. All human research protocols received approval from the University of California, San Francisco Committee on Human Research (UCSF IRB 10-02246). Results were reported as concentrations of free, conjugated, and total analyte in each sample.

## Results and discussion

### Development of a direct analytical method

To analyze representative chemicals from three different classes of EDCs, we developed and validated a direct LC–MS/MS assay to simultaneously quantify BPS, PrP, MBP, and their major phase II conjugates in human urine. From an analytical perspective, these targets constitute a stringent test because they span several subclasses of EDCs, common environmental contaminants that are typically quantified using indirect, enzyme-catalyzed hydrolysis methods. Importantly, the extent of hydrolysis has not been systematically evaluated for any of the three analytes.

**FIGURE 1:**
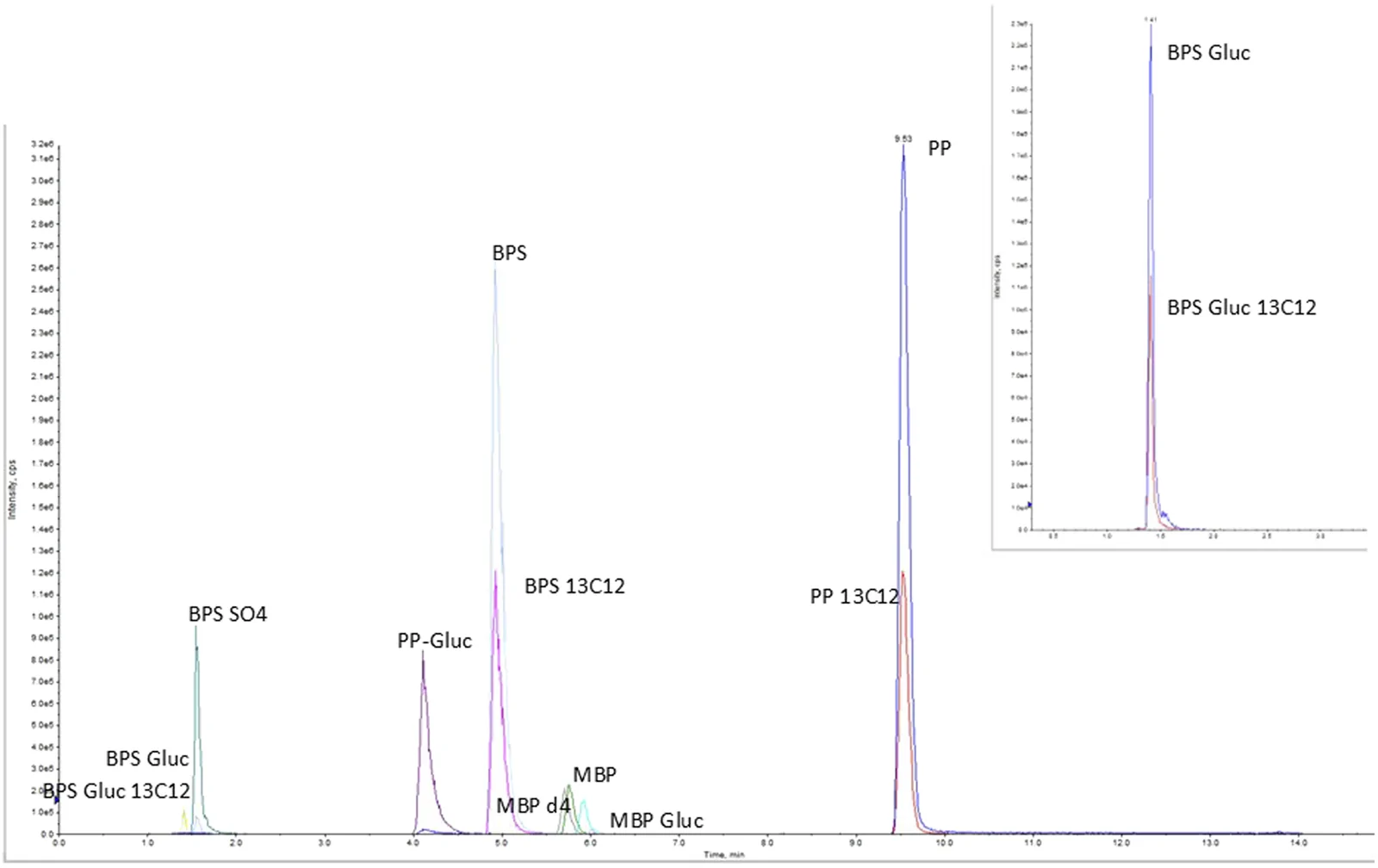
Representative chromatogram of the validated panel for bisphenol S (BPS), propylparaben (PrP), monobutyl phthalate (MBP) and their metabolites.

Despite structural heterogeneity, the selected phenolic and phthalate metabolites share ionizable acidic functionalities. This enabled their consolidation into a single negative-ion ESI method, minimizing instrument time and inter-method variability. For each analyte, a quantifier transition was used for quantification while a concurrently monitored qualifier transition served as a confirmatory ion. Consistent with current FDA bioanalytical guidance for MRM-based assays, confirmation of the analyte was based on a transition ion ratio within ±20% of the mean calibrator ratio. Additionally, to reduce reliance on MRM selectivity alone for specificity, a C18 reversed-phase gradient was used to resolve parent compounds and their glucuronide and sulfate conjugates across a broad polarity window. As shown in Figure 1, this provided baseline separation of closely eluting species.

To maximize removal of proteins and phospholipids, a polymer-based SPE sorbent was used to limit ion suppression and provide uniform retention across analytes with divergent hydrophobicity. Although isotopically labeled conjugated standards were not commercially available for several metabolites (i.e., MBP-glucuronide, BPS-sulfate, PrP-glucuronide), structurally or chromatographically aligned surrogates afforded stable isotope dilution for all targets, yielding reproducible peak area ratios and supporting quantitative robustness in the absence of ideal internal standards.

### Method validation

All validation parameters satisfied or exceeded contemporary bioanalytical guidance for chromatographic assays, including selectivity, specificity, linearity, precision, accuracy, recovery, matrix effect, and carryover (Food and Drug Administration, 2018). Method validation was conducted using synthetic human urine (SHU) as the primary matrix blank. For non-persistent EDCs that are ubiquitously present in the general population, such as BPS, PrP, and MBP, it is practically impossible to obtain pooled human urine that is truly free of all target analytes and thus suitable as an analyte-free blank. SHU provides a compositionally defined, low-background matrix with stable properties over time, that enabled us to isolate analytically driven differences between direct and indirect workflows while still approximating typical urinary ionic strength and major solute composition.

Matrix blanks and mixtures of structurally related EDCs produced no measurable signal at the MRM transitions of interest, indicating high method specificity and negligible qualitative interference from co-occurring contaminants. Calibration curves for all analytes were constructed using linear regression, with 1/x weighting applied, yielding mean r^2^ values of 0.997 or higher (n = 5), across 3-4 orders of magnitude and supporting reliable back-calculation across the entire biomonitoring range. LOD and LLOQ values (Table 2) were well aligned with, and in most cases lower than, concentrations typically encountered in population-based urine surveys, with LODs ≤0.1 ng/mL for all analytes except BPS glucuronide, thereby exceeding the sensitivity of current NHANES protocols and most published indirect assays.

**TABLE 2:** Measured retention time and sensitivity, and quality control levels used for each analyte.*

Analyte	RT (min)	LOD (ng/mL)	LLOQ (ng/mL)	ULOQ (ng/mL)	QC level (ng/mL)	
					Low	High
Bisphenol S	4.91	0.02	0.1	100	0.15	25
Bisphenol S sulfate	1.54	0.05	0.1	250	0.15	100
Bisphenol S glucuronide	1.41	0.25	0.5	250	0.75	100
Mono butyl phthalate	5.76	0.1	0.25	250	1.5	100
Mono butyl phthalate glucuronide	5.91	0.1	0.1	250	0.75	100
Propyl paraben	9.52	0.02	0.05	100	0.15	25
Propyl paraben glucuronide	4.10	0.02	0.05	250	0.15	100

* RT, retention time; LOD, limit of detection; LLOQ, lower limit of quantification; ULOQ, upper limit of quantification; QC, quality control.

Isotope-dilution quantification demonstrated excellent repeatability and trueness. Intra- and inter-day QC results showed precision ≤7% CV and absolute relative error ≤10% at both concentration levels, comfortably within the ±15% limits recommended for bioanalytical methods and consistent with best-practice targets for minimized run rejection rates (Food and Drug Administration, 2018). Re-injection experiments produced CVs <7% and RE <9%, demonstrating extract stability and instrumental reproducibility during routine batch operation (Table 3).

**TABLE 3:** Precision and accuracy for each analyte at two quality control levels using five replicates at each run (within run) in three separate instances (between runs).

Analyte	Method parameter	Within-run		Between-run		Reinjection reproducibility	
		Low	High	Low	High	Low	High
Bisphenol S	Precision (%CV)	5%	2%	3%	3%	6%	2%
	Accuracy (%RE)	2%	6%	3%	4%	3%	3%
Bisphenol S sulfate	Precision (%CV)	5%	3%	4%	4%	7%	5%
	Accuracy (%RE)	6%	3%	5%	4%	5%	1%
Bisphenol S glucuronide	Precision (%CV)	0%	0%	4%	5%	7%	2%
	Accuracy (%RE)	3%	3%	4%	2%	4%	1%
Mono butyl phthalate	Precision (%CV)	1%	1%	1%	6%	2%	2%
	Accuracy (%RE)	4%	1%	5%	2%	3%	6%
Mono butyl phthalate glucuronide	Precision (%CV)	9%	4%	7%	6%	7%	2%
	Accuracy (%RE)	2%	2%	3%	2%	6%	4%
Propyl paraben	Precision (%CV)	2%	10%	1%	5%	5%	2%
	Accuracy (%RE)	4%	1%	4%	2%	3%	7%
Propyl paraben glucuronide	Precision (%CV)	3%	1%	3%	3%	5%	4%
	Accuracy (%RE)	4%	5%	4%	5%	8%	9%

SPE recoveries were high and consistent across analytes, with recovery CVs ≤13% at both QC levels, while matrix effects in synthetic urine were generally modest; signal suppression was most evident for PrP glucuronide but remained controlled, with variability in matrix factor ≤8% CV (Table 4). As recommended in current guidance, the evaluation emphasized recovery and matrix effects precision rather than absolute values (Food and Drug Administration, 2018), and no significant carryover was detected in blank injections following high QC samples. Collectively, these metrics confirm that the direct LC–MS/MS method is analytically fit-for-purpose for trace-level biomonitoring.

**TABLE 4:** Recovery and matrix effect of the analytes at two quality control levels.

Analyte	Method parameter	Matrix effect		Recovery	
		Low	High	Low	High
Bisphenol S	% Average	111%	104%	93%	102%
	Precision (%CV)	3%	4%	4%	2%
Bisphenol S sulfate	% Average	92%	113%	94%	94%
	Precision (%CV)	5%	1%	4%	5%
Bisphenol S glucuronide	% Average	90%	102%	93%	100%
	Precision (%CV)	4%	4%	4%	5%
Mono butyl phthalate	% Average	100%	99%	99%	99%
	Precision (%CV)	1%	3%	1%	6%
Mono butyl phthalate glucuronide	% Average	111%	106%	102%	99%
	Precision (%CV)	8%	4%	13%	6%
Propyl paraben	% Average	91%	96%	99%	104%
	Precision (%CV)	4%	5%	4%	6%
Propyl paraben glucuronide	% Average	85%	75%	102%	93%
	Precision (%CV)	4%	5%	3%	13%

### The efficacy of the indirect method across a range of concentrations

**FIGURE 2:**
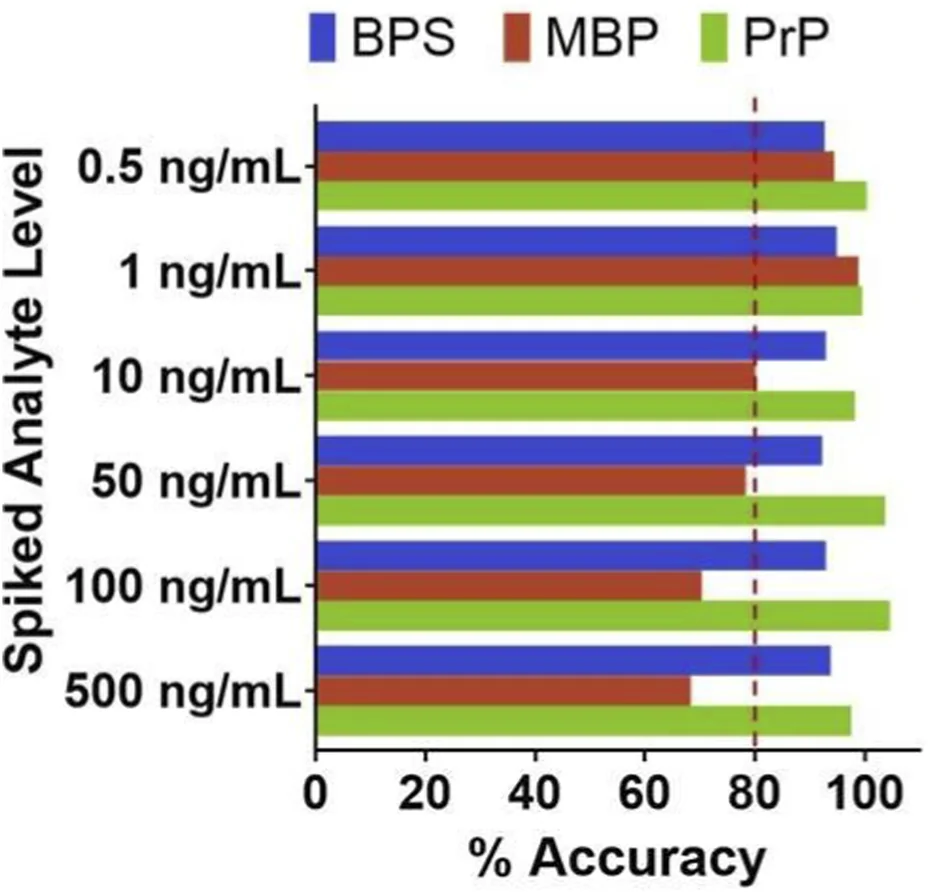
Comparative accuracy of the indirect method for bisphenol S (BPS), propylparaben (PrP) and monobutyl phthalate (MBP) using synthetic human urine samples with six different concentrations of BPS glucuronide, PrP glucuronide and MBP glucuronide. The red broken line represents the cutoff commonly used for accuracy.

The availability of a rigorously validated direct assay enabled a mechanistic evaluation of bias inherent to conventional indirect, enzyme-based deconjugation workflows. Using synthetic urine, we tested the ability of indirect analysis to recover metabolites of the three EDCs across a broad concentration range. Spiked levels of the three glucuronidated EDCs in synthetic urine demonstrated differences among the analytes. Indirect measurements were in close agreement with direct totals for BPS and PrP (≈90–102% of nominal), indicating that hydrolysis was analytically adequate for these phenolic substrates over the tested range. In contrast, MBP exhibited systematic, concentration-dependent negative bias, with apparent recovery decreasing from roughly quantitative at 0.5 ng/mL to approximately 65% at 500 ng/mL (Figure 2). This pattern mirrors, albeit to a lesser extent, the previously documented underestimation of BPA when comparing direct and indirect measurements (Gerona et al., 2020), underscoring that hydrolysis efficiency cannot be assumed to be uniform across EDC classes.

Although indirect methods are commonly used to measure EDCs in human biospecimens, mechanistic evaluations of the enzyme-based deconjugation workflows used in these methods are rarely conducted. Our investigation of the accuracy of indirect methods for analyzing three EDCs in spiked synthetic urine samples demonstrates variability in their ability to faithfully capture metabolites. These results extend our prior work on BPA (Gerona et al., 2020), demonstrating that under-recovery is not a rare anomaly with use of indirect analysis but can occur for other non-persistent EDCs. This is exemplified by MBP, which exhibited a concentration-dependent effect, even when phenolic analytes such as BPS and PrP were quantitatively captured in the same workflow.

The Achille’s heel of indirect approaches is their reliance on the deconjugation of 4-methylumbelliferone glucuronide as a surrogate to monitor enzymatic hydrolysis by H. pomatia β glucuronidase. This assumes shared equivalent enzyme–substrate kinetics. Given the basic principle of specificity in substrate-enzyme kinetics, however, the catalytic efficiency and extent of hydrolysis of 4-methylumbelliferone glucuronide by H. pomatia β glucuronidase cannot be extrapolated to structurally distinct analyte conjugates without experimental verification. To date, published indirect methods have not provided a systematic, analyte-specific characterization of these enzyme-substrate interactions, largely because such studies require direct LC-MS/MS quantification of both free and conjugated species-a strategy that even large biomonitoring programs such as NHANES have not implemented routinely, despite the availability of reference standards for several EDC metabolites. Our present data provide experimental evidence that indirect assays can yield analyte-specific and concentration-dependent underestimation of urinary MBP. This concentration-dependent negative bias implies that the apparent performance of an indirect assay at low concentrations (e.g., near LODs used in population surveys) cannot be extrapolated to the upper tail of exposure distributions, where risk assessments often focus.

### Using direct analysis to assess the ability of indirect methods to capture metabolites in human urine

Application of the validated direct method to 30 archived second-trimester maternal urine samples demonstrated its suitability for complex, real-world matrices and enabled the first simultaneous determination of free and conjugated forms of BPS, PrP, and MBP in a pregnant cohort. Summarized results are presented in Tables 5, 6. All target analytes were detectable in nearly all samples. Glucuronide conjugates accounted for more than 90% of the total signal, consistent with established phase II biotransformation pathways for these compounds (Abbas et al., 2010; Durcik et al., 2022; Frederiksen et al., 2007; Vom Saal et al., 2014). Geometric mean total concentrations for BPS, PrP, and MBP (18.1, 30.3, and 41.2 ng/mL, respectively) exceeded NHANES estimates based on indirect analysis of urine for comparable years by factors of roughly 22, 3, and 5 (Table 7) (Jiang et al., 2023), indicating that a combination of assay bias, cohort characteristics, and metabolic state may substantially shift apparent exposure distributions relative to national reference values.

**TABLE 5:** Levels of bisphenol S, propylparaben, monobutyl phthalate and their metabolites (ng/mL) in 30 second-trimester maternal urine samples.

Sample	Avg BPS	Avg BPS sulfate	Avg BPS gluc	BPS total	Avg MBP	Avg MBP gluc	MBP total	Avg PrP	Avg PrP gluc	PrP total	Avg creatinine (mg/dL)
1	2.40	0.91	31.56	**21.66**	1.12	80.91	**46.26**	34.27	356.21	**214.41**	209.70
2	0.40	<LOD	42.31	**25.28**	0.64	24.10	**14.09**	5.80	123.14	**68.08**	91.39
3	0.47	<LOD	12.99	**8.11**	0.17	37.68	**21.19**	0.10	31.90	**16.24**	104.53
4	0.50	<LOD	36.50	**21.97**	1.16	88.89	**50.75**	1.79	134.38	**69.74**	122.94
5	0.76	1.06	17.03	**11.57**	6.98	236.86	**139.12**	11.86	228.35	**127.34**	129.41
6	0.76	<LOD	14.90	**9.52**	7.96	153.89	**93.81**	5.84	110.26	**61.60**	110.85
7	0.91	<LOD	17.34	**11.11**	1.73	64.36	**37.64**	0.89	80.04	**41.37**	111.64
8	0.43	<LOD	16.63	**10.21**	0.20	40.04	**22.53**	0.07	10.20	**5.23**	140.94
9	0.40	<LOD	25.35	**15.31**	20.32	634.14	**374.10**	0.06	3.10	**1.62**	82.16
10	0.39	<LOD	23.46	**14.19**	<LOD	45.19	**25.21**	0.69	51.62	**26.79**	88.05
11	0.78	0.26	7.89	**5.61**	0.70	22.08	**13.01**	1.85	11.78	**7.81**	67.29
12	0.93	1.38	46.08	**29.08**	1.11	63.33	**36.44**	0.60	95.01	**48.64**	223.25
13	0.79	0.65	29.12	**18.40**	<LOD	23.13	**12.90**	0.07	133.30	**67.48**	102.95
14	0.86	0.50	55.89	**34.11**	0.19	46.40	**26.08**	3.61	256.53	**133.34**	192.30
15	0.47	<LOD	33.27	**20.04**	2.25	235.48	**133.62**	0.15	72.30	**36.71**	194.87
16	0.86	<LOD	36.13	**22.10**	2.57	29.03	**18.76**	2.34	59.69	**32.53**	98.85
17	0.54	0.29	25.12	**15.53**	1.01	108.03	**61.27**	<LOD	8.38	**4.24**	165.10
18	1.28	0.74	8.23	**6.68**	7.69	96.63	**61.60**	0.57	29.35	**15.41**	74.37
19	0.60	<LOD	33.22	**20.13**	0.17	91.98	**51.49**	<LOD	13.25	**6.70**	146.87
20	0.56	<LOD	57.33	**34.28**	1.31	153.67	**87.04**	0.10	409.58	**207.23**	130.04
21	0.32	1.36	49.61	**30.53**	2.59	24.31	**16.15**	<LOD	9.85	**4.98**	100.82
22	0.36	<LOD	39.07	**23.34**	0.69	72.91	**41.37**	0.12	15.78	**8.10**	129.30
23	0.41	1.82	45.24	**28.40**	1.53	64.97	**37.77**	1.33	67.71	**35.58**	124.13
24	0.73	0.23	74.38	**44.64**	3.05	127.23	**74.03**	0.36	153.13	**77.80**	251.35
25	0.37	0.27	44.18	**26.56**	0.52	44.26	**25.21**	4.22	556.38	**285.59**	107.51
26	3.92	3.61	38.35	**29.21**	16.39	174.94	**113.99**	0.16	27.13	**13.88**	79.81
27	3.59	<LOD	16.64	**13.38**	5.91	22.49	**18.45**	3.07	53.46	**30.11**	101.37
28	0.66	0.16	35.41	**21.60**	0.14	47.08	**26.41**	<LOD	64.87	**32.81**	234.84
29	0.90	<LOD	41.29	**25.18**	1.66	183.96	**104.29**	0.05	39.42	**19.99**	233.58
30	0.27	<LOD	21.11	**12.68**	<LOD	37.42	**20.87**	0.37	143.98	**73.18**	48.57

The values in bold represent the total values for each analyte.

**TABLE 6:** Descriptive statistics of measured bisphenol S, propylparaben, monobutyl phthalate and their metabolites in 30 second-trimester maternal urine.

Parameter	BPS	BPS sulfate	BPS gluc	BPS total	MBP	MBP gluc	MBP total	PrP	PrP gluc	PrP total
Mean*	0.88	0.94	32.52	**20.35**	3.32	102.51	**60.18**	3.09	111.67	**59.15**
Geometric mean*	0.68	0.64	28.49	**18.12**	1.40	69.94	**41.21**	0.69	57.61	**30.34**
Range*	0.27–3.92	0.16–3.61	7.89–74.38	**5.61–44.64**	0.14–20.32	22.08–634.14	**12.90–374.10**	0.05–34.27	3.10–556.38	**1.62–285.59**
Detection frequency	30	14	30	**30**	27	30	**30**	26	30	**30**

* All levels are in ng/mL.

The values in bold represent the total values for each analyte.

**TABLE 7:** Indirect measurements of bisphenol S, propylparaben, and monobutyl phthalate in relevant cohorts.*

Source	N	BPS**			MBP**			PrP**			Cohort
		Mean	Geo mean	Median	Mean	Geo mean	Median	Mean	Geo mean	Median	
Copenhagen, 2022 (Bräuner et al., 2022)	107	—	—	—	10.7	—	8.19	10.8	—	2.51	Pregnant women
Spain, 2019 (Sanchis et al., 2019)	15	1.22	0.22	0.07	—	—	—	0.59	0.18	0.07	Lactating women
Australia, 2016 (Heffernan et al., 2016)	30	—	—	—	13.6	10.9	11.8	—	—	—	Pregnant women
China, 2020 (Zhang et al., 2020)	15	—	0.05	—	—	—	—	—	—	—	Pregnant women
United States of America, 2017 (Pollack et al., 2016)	143	—	—	—	—	—	—	—	12.5	—	Premeno—pausal women
United States of America, 2015 (Pycke et al., 2015)	181	—	—	—	—	—	—	171	—	75.3	Pregnant women
United States of America, 2012 (Liao et al., 2012)	31	1.120	0.299	0.263	—	—	—	—	—	—	Adults
United States of America, 2014 (Zhou et al., 2014)	100	—	—	0.13	—	—	—	—	—	—	Adults
United States of America, 2008 (Adibi et al., 2008)	246	—	—	—	—	37.5	—	—	—	—	Pregnant women
United States of America, 2018 (Zhou et al., 2018)	115	—	—	—	42.8	—	25.9	—	—	—	Pregnant women
NHANES 2007–2008 (Jiang et al., 2023)	1,310	—	—	—	—	19.3	—	—	18.4	—	Women
NHANES 2009–2010 (Jiang et al., 2023)	1,350	—	—	—	—	14.7	—	—	16.9	—	Women
NHANES 2011–2012 (Jiang et al., 2023)	1,230	—	—	—	—	7.14	—	—	11.6	—	Women
NHANES 2013–2014 (Jiang et al., 2023)	1,398	—	0.40	—	—	8.76	—	—	12.2	—	Women
NHANES 2015–2016 (Jiang et al., 2023)	1,344	—	0.44	—	—	9.78	—	—	8.77	—	Women
NHANES 2017–2018 (Jiang et al., 2023)	1,399	—	—	—	—	8.34	—	—	—	—	Women
Our Study, 2011–2014	**30**	**20.35**	**18.12**	**20.87**	**60.18**	**41.21**	**37.71**	**59.15**	**57.61**	**34.20**	Pregnant women

* Geo Mean, Geometric mean; BPS, bisphenol S; MBP , monobutyl phthalate; PrP, propylparaben.

** All levels are in ng/mL.

The values in bold represent the values obtained in our current study.

Findings from maternal urine differ in detail from those in synthetic urine but are consistent in their broader implications. The MBP results are directionally consistent with the under-recovery results of analyses of spiked synthetic urine (Figure 2), supporting the conclusion that hydrolysis-based protocols underreport this phthalate glucuronide at higher concentrations. For BPS and PrP, where direct and indirect methods agreed in synthetic urine, elevated urinary levels suggest that additional factors beyond assay bias are involved. In particular, synthetic human urine may not fully reproduce the complexity of the maternal urine matrix, and such differences can affect key method parameters, including deconjugation efficiency in the indirect method, steps that are bypassed in our direct approach, and thereby contribute to discrepancies between matrices. Elevated urinary levels in pregnant women may be attributed to physiology and exposure rather than solely an assay bias, e.g., pregnancy-related changes in renal handling, urine composition, and conjugation capacity (Soma-Pillay et al., 2016), as well as product use patterns documented in previous studies of parabens and bisphenols during pregnancy (Adibi et al., 2008; Pycke et al., 2015). The cohort’s predominantly low socioeconomic status likely further amplifies exposure, consistent with literature demonstrating higher concentrations of non-persistent EDCs among socioeconomically disadvantaged populations (Montazeri et al., 2019; Polinski et al., 2018). In summary, the magnitude and pattern of the observed differences suggest that both analytical and biological factors contribute to the actual maternal urine samples.

Collectively, our findings argue for a shift in validation practices from generic surrogate-based checks toward analyte-resolved evaluation of hydrolysis efficiency across relevant concentrations, ideally using direct LC–MS/MS quantification of both conjugated and deconjugated species as an internal benchmark. In practical terms, laboratories that continue to use indirect methods should, at a minimum, incorporate periodic paired direct–indirect comparisons for representative panels of conjugates, rather than relying solely on 4-methylumbelliferyl glucuronide or other model substrates as process controls. Our work also underscores the value of expanding the commercial availability of isotope-labeled conjugated standards for phthalates, parabens, and bisphenols: these materials would enable more rigorous isotope-dilution calibration of conjugates themselves, facilitate mechanistic kinetic studies with different β-glucuronidase preparations, and support cross-laboratory harmonization of direct methods. Finally, the discrepancy between synthetic and maternal urine findings highlights the need to incorporate physiologic states and matrix diversity into method development; pregnancy-related changes in urine composition, protein and organic acid content, and pH could modulate both enzymatic hydrolysis and ionization efficiency, so validation solely in synthetic matrices risks underestimating real-world variability in performance.

### Limitations and future directions

From an analytical standpoint, a key limitation of our study is reliance on synthetic urine for much of the formal validation and for the indirect-versus-direct comparison. Although synthetic matrices can be tuned to approximate average ionic strength, pH, and major solute composition, they do not capture the full spectrum of endogenous organic constituents that may influence extraction efficiency, ionization, and enzymatic hydrolysis. This is of particular concern during pregnancy, a time of dramatic physiological change. Ongoing work is therefore extending paired direct and indirect measurements of urine from pregnant individuals, which will allow matrix-matched assessment of hydrolysis efficiency and matrix effects and clarify whether the biases observed for MBP can be generalized across populations. More importantly, these data will determine if matrix effects in pregnant urine differ significantly from those in synthetic human urine thereby explaining the difference in observations of bias in BPS and PrP. A second limitation is the cohort’s geographic and demographic restrictions, which limit extrapolation of concentration distributions but do not affect the fundamental analytical conclusions regarding method performance. Finally, the absence of a commercial PrP-sulfate standard precluded inclusion of this metabolite; targeted synthesis and characterization of additional conjugated reference materials would enable more exhaustive coverage of paraben and bisphenol biotransformation products in future LC-MS/MS panels.

### Implications

The findings have direct implications for the design and interpretation of national biomonitoring efforts. From a policy perspective, underestimation of urinary MBP and potentially other phthalate metabolites by indirect methods can propagate through the entire risk-assessment pipeline, leading to biased exposure distributions, misclassification of individuals across exposure categories, and attenuation of exposure–response estimates in epidemiologic analyses. Because urinary metabolite data from NHANES and similar surveys are widely used to derive reference doses, to evaluate population-level trends, and to inform regulatory actions on phthalates and other EDCs, a systematic low bias in measured concentrations implies that current “background” levels may in fact be higher than reported, especially in subgroups with elevated exposure such as pregnant people, children, and socioeconomically disadvantaged communities. This has several knock-on effects: margins of exposure calculated relative to toxicological points of departure may be overestimated; the apparent lack of clear thresholds or the shape of nonmonotonic dose-response relationships could be mischaracterized; and the effectiveness of regulatory interventions judged by temporal trends in biomonitoring data may be overstated if declining trends partly reflect analytical artifacts rather than genuine exposure reductions.

Direct methods that resolve free and conjugated species and achieve sub-ng/mL sensitivity, therefore, offer more than incremental analytical refinement; they provide a pathway to recalibrating existing exposure databases and to designing future biomonitoring campaigns aligned with contemporary risk-assessment needs for EDCs. For agencies and consortia overseeing large biomonitoring programs, our findings support several pragmatic steps: (1) incorporating direct LC–MS/MS panels for key non-persistent EDC metabolites, at least in a representative subset of samples, to empirically quantify indirect-method bias; (2) revisiting historical datasets using correction factors or reanalysis of stored samples where feasible; and (3) explicitly accounting for method-related uncertainty and potential low bias in exposure reconstruction and health-based guidance values. More broadly, these data reinforce calls within the evolving risk-assessment literature to integrate advances in analytical chemistry into regulatory decision-making more rapidly, particularly for chemicals like phthalates and bisphenols that exhibit complex, potentially non-threshold dose–response behavior and disproportionate burdens in vulnerable populations.

## Conclusion

This study establishes a rigorously validated, direct LC-MS/MS platform for the concurrent quantification of BPS, PrP, MBP, and their major urinary conjugates, emphasizing analytical performance criteria central to high-quality biomonitoring. The method demonstrates sub-ng/mL sensitivity for most analytes, excellent linearity over several orders of magnitude, and robust precision, accuracy, and specificity compatible with contemporary bioanalytical standards. By explicitly characterizing matrix effects, extraction recovery, and instrumental reproducibility, the assay is shown to be analytically fit-for-purpose for the trace-level assessment of non-persistent EDCs in complex biological matrices such as urine.

Direct comparison with conventional hydrolysis-based workflows shows that analytical bias is strongly analyte-dependent, with enzymatic deconjugation yielding a substantial underestimation of MBP at higher concentrations, while performing adequately for BPS and PrP. These results underscore that indirect methods cannot be assumed to provide uniform quantitative reliability across structurally diverse glucuronides and sulfates, particularly when enzyme kinetics and matrix composition vary. Adoption of direct, isotope-dilution LC-MS/MS strategies that resolve free and conjugated species will be essential for minimizing exposure misclassification, improving comparability across studies, and strengthening the evidentiary basis for risk assessment of non-persistent EDCs. In large biomonitoring programs, incorporating direct panels would also enable empirical quantification and correction of hydrolysis-related bias, thereby aligning exposure estimates more closely with the true internal dose distributions that underpin regulatory decisions.

## Data Availability

The original contributions presented in the study are included in the article, further inquiries can be directed to the corresponding author.
